# Photosynthetic Performance of Oil Palm Genotypes under Drought Stress

**DOI:** 10.3390/plants13192705

**Published:** 2024-09-27

**Authors:** Carmenza Montoya, Edison Daza, Fernan Santiago Mejía-Alvarado, Arley Fernando Caicedo-Zambrano, Iván Ayala-Díaz, Rodrigo Ruiz-Romero, Hernán Mauricio Romero

**Affiliations:** 1 Oil Palm Biology and Breeding Research Program, Colombian Oil Palm Research Center—Cenipalma, Bogotá 11121, Colombia; cmontoya@cenipalma.org (C.M.); edaza@cenipalma.org (E.D.); fmejia@cenipalma.org (F.S.M.-A.); acaicedo@cenipalma.org (A.F.C.-Z.); iayala@cenipalma.org (I.A.-D.); rruiz@cenipalma.org (R.R.-R.); 2 Department of Biology, Universidad Nacional de Colombia, Bogotá 11132, Colombia

**Keywords:** oil palm, drought stress, photosynthesis, gas exchange

## Abstract

Water deficiency and potential drought periods could be important ecological factors influencing cultivation areas and productivity once different crops are established. The principal supply of vegetable oil for oil crops is oil palm, and new challenges are emerging in the face of climatic changes. This study investigated the photosynthetic performance of 12 genotypes of *Elaeis* exposed to drought stress under controlled conditions. The assay included genotypes of *Elaeis guineensis, Elaeis oleifera,* and the interspecific O×G hybrid (*E. oleifera* × *E. guineensis*). The principal results showed that the *E. guineensis* genotype was the most efficient at achieving photosynthesis under drought stress conditions, followed by the hybrid and *E. oleifera* genotypes. The physiological parameters showed good prospects for vegetal breeding with different O×G hybrids, mainly because of their ability to maintain the equilibrium between CO_2_ assimilation and stomatal aperture. We validated 11 genes associated with drought tolerance, but no differences were detected. These results indicate that no allelic variants were represented in the RNA during sampling for the validated genotypes. In conclusion, this study helps to define genotypes that can be used as parental lines for oil palm improvement. The gas exchange data showed that drought stress tolerance could define guidelines to incorporate the available genetic resources in breeding programs across the early selection in nursery stages.

## 1. Introduction

Plants adapt to various environmental conditions by employing physiological strategies to survive unfavorable biotic and abiotic stresses. Water deficiency and potential drought periods are significant ecological factors influencing cultivation areas and crop productivity. Currently, palm oil is the leading source of vegetable oil, with an annual production of 80 million metric tons (MMT), followed by soybean oil (65 MMT) and rapeseed oil (34 MMT) [1]. This underscores the importance of understanding the performance of these crops during unpredictable drought periods, especially in the face of climate challenges or in regions with limited water supply.

The species that produce palm oil worldwide are *Elaeis guineensis* Jacq, which initially came from the African continent; however, its wild relative *Elaeis oleifera* (Kunth) Cortés, which is endemic to Central and South America, lacks commercial prospects but has some agronomical traits that are useful for introgression to commercial African oil palm cultivars [2]. With these two species, it is possible to generate the interspecific O×G hybrids (*Elaeis oleifera × Elaeis guineensis*) which are critical in Latin America, especially in the Colombian and Ecuadorian oil palm industries, due to its tolerance to bud rot (BR) disease, one of the most devastating diseases affecting oil palm in the Americas [3,4]. Additionally, the O×G genotypes are relevant since it is plausible to reach more than 10 tons per hectare per year of high-oleic palm oil (HOPO) using naphthalene acetic acid (NAA) to produce parthenocarpic fruits without increasing the planted oil palm area [5,6]. 

In Colombia, climate change constrains the oil palm industry due to water scarcity in some cultivated regions. Genetic resources are the key to overcoming limiting problems in agricultural systems, where drought responses could vary depending on the genetic background. The drought tolerance of commercial cultivars has been studied to characterize the response to a limiting abiotic factor that may not have been considered in the original breeding programs [7,8]. The results obtained for the O×G hybrid and its physiological response to water deficit [9] included the performance of O×G U1937, which showed a tolerance profile under water deficit conditions. 

Physiological variables, such as the net photosynthetic rate (A), stomatal conductance (gs), transpiration rate (E), instantaneous leaf-level water use efficiency (WUE), leaf-to-air vapor pressure deficit (Δw), and leaf water potential (ΨLeaf), are crucial to understanding the mechanisms that permit the regulation and efficient use of water. The above directly correlates with the stomatal opening time and stomatal resistance because while plants absorb CO_2_ for photosynthesis, water is lost through transpiration, with variable intensity depending on the stomatal conductance and the water potential gradient between the leaf surface and the atmosphere [10,11]. Photosynthetic efficiency has been studied to elucidate part of the mechanisms of tolerance to drought stress in oil palm [7,8,9,12,13,14]. Monitoring gas exchange data to understand drought tolerance phenomena is a current tool in breeding programs for diverse crops, such as potato [15], tomato [16], maize [17], hemp [18], teak [19], and soybean [20,21].

On the other hand, plant breeding studies supported by genomics or transcriptome analysis increase the opportunity to identify the critical genes involved in resistance to abiotic stresses such as water scarcity [22,23,24]. Additionally, the development of molecular marker-assisted selection (MAS) methods requires the study of molecular mechanisms and their applications in the early stages of genotype selection or constructing genomic selection models to estimate breeding values [25,26,27]. Transcriptome analyses of Cenipalma [28] in *E. guineensis* under drought stress revealed that gene families participate in the tolerance to abiotic stress, specifically drought. Our previous results were consistent with those of other crops exposed to similar conditions, so we validated 11 genes by RT-qPCR and conventional PCR in the genotypes under observation in this assay. The principal genes identified corresponded to genes or gene families such as *SWEET* sucrose transporters [29,30], the *XTH* gene family (xyloglucan transglycosylase/hydrolase) [31], elongation factor 1 [32], *WRKY* transcription factors [33], *GOLS1*-galactactinol synthase 1 [34,35], *GAPDH*-glyceraldehyde-3-phosphate dehydrogenase [25], the *NAC* transcription factor family [36], *MCTPs*-multiple C2 domain and transmembrane region proteins [37], the *CCR4*-association factor 1 gene family [38], the *PPR*-pentatricopeptide repeat gene family_At5g39980 [39], the *nsLTP*-nonspecific lipid transfer protein family [23,40], the leucine-rich repeat receptor-like serine [22], and the *XYL2*-beta-xylosidase alpha-L-arabinofuranosidase 2-like [41].

In this research, we evaluated the response of African oil palm (*Elaeis guineensis* Jacq), American oil palm (*Elaeis oleifera* (Kunth) Cortés), and the interspecific O×G hybrid (*E. oleifera × E. guineensis*) to drought stress under mesh house conditions to understand the photosynthetic performance of oil palm genotypes under drought stress and the physiological and genetic mechanisms involved to support O×G and D×P (*dura* × *pisifera*) breeding programs. 

## 2. Results

### 2.1. Physiological Parameters

The descriptive analysis revealed substantial changes in the physiological parameters between soil treatments with the optimal amount of moisture (well-watered treatment) and drought stress (Table 1). 

The transpiration rate (E) decreased from 3.84 to 0.713 mmol H_2_O m^−2^s^−1^, the photosynthetic rate (A) decreased from 10.4 to 3.80 μmol CO_2_ m^−2^s^−1^, and the stomatal conductance (gs) decreased from 0.244 to 0.0384 mmol H_2_O m^−2^s^−1^. The leaf–air vapor pressure deficit (Δw) increased slightly from 1.61 to 1.87 kPa. In contrast, the instantaneous water use efficiency (WUE) notably rose from 2.79 to 6.49 µmol CO_2_/mmol H_2_O, and the leaf water potential (Ψleaf) decreased drastically from −0.07 MPa to −1.0 MPa, indicating severe water stress in plants under drought stress. These changes indicate a strong response of plants to water stress, with a marked reduction in gas exchange and an increase in water use efficiency. 

**Figure 1:**
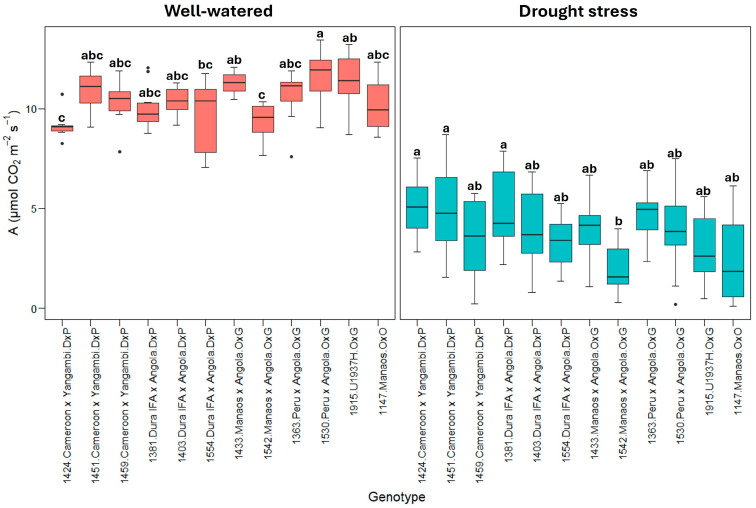
A: Net photosynthetic rate (µmol CO_2_ m^−2^s^−1^). Treatment by genotype. Tukey’s mean comparison test shows that values with the same letter do not present statistically significant differences (*p* < 0.05).

Figure 1 shows the net photosynthetic rate (A). All genotypes significantly reduced the photosynthetic rates under drought stress, but there was variability among them. Some genotypes, especially *E. guineensis* (DxP), showed a greater capacity to maintain photosynthetic rates under stress. The *E. guineensis* genotypes generally presented a lower reduction, from an average of approximately 10.0 ± 0.9 µmol CO_2_ m^−2^s^−1^ in the well-watered treatment to 4.2 ± 1.6 µmol CO_2_ m^−2^s^−1^ in the drought stress treatment, which represents a decrease of roughly 60%. However, in the O×G hybrid and *E. oleifera* genotypes, the reduction in the photosynthetic rate was more remarkable, from 10.7 ± 1.3 µmol CO_2_ m^−2^s^−1^ in the hybrid O×G and 10.3 ± 0.9 µmol CO_2_ m^−2^s^−1^ in the well-watered *E. oleifera* to 3.5 ± 1.6 and 2.9 ± 1.3, respectively, under drought stress, which represents decreases close to 70% (Figure 1).

Significant contrasts were observed among the twelve genotypes evaluated, even within the same genetic origins. For instance, the 1424 Cameroon × Yangambi and 1381 Dura IFA × Angola genotypes exhibited the most minor reductions in the photosynthetic rate, with decreases of 47% and 50%, respectively. In comparison, the 1459 Cameroon × Yangambi (67%) and 1554 Dura IFA × Angola (66%) genotypes, along with the *E. guineensis* genotype, showed the most significant reductions in photosynthesis due to water deficit.

Within the O×G hybrid group (Figure 1), the 1363 Peru × Angola genotype exhibited the smallest decrease in photosynthesis under water deficit conditions, with a reduction from 10.2 ± 1.7 µmol CO_2_ m^−2^s^−1^ under well-watered conditions to 5.2 ± 1.2 µmol CO_2_ m^−2^s^−1^ under drought stressa 50% decrease. In contrast, the O×G 1542 Manaos × Angola and 1915 (U1937H) genotypes exhibited photosynthesis values between 2.0 and 3.2 µmol CO_2_ m^−2^s^−1^ under drought stress, representing decreases of 78% and 72%, respectively, from the rates observed under optimal soil moisture conditions.

**Figure 2:**
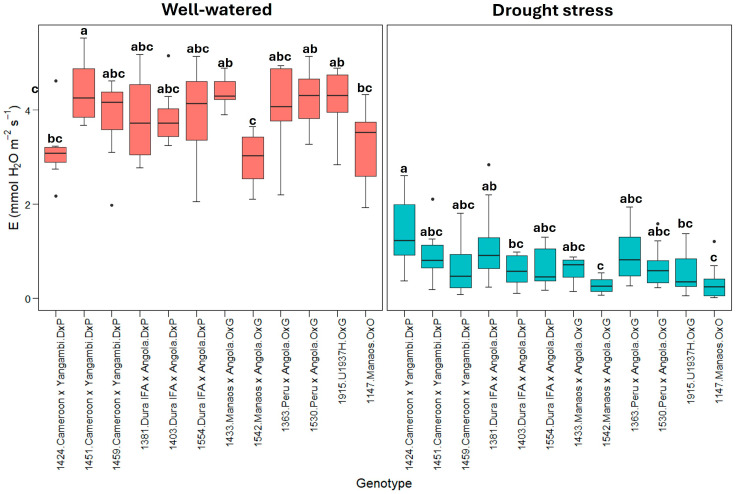
E: transpiration rate (mmol H_2_O m^−2^s^−1^). Treatment by genotype. Tukey’s mean comparison test shows that values with the same letter do not present statistically significant differences (*p* < 0.05).

The box plot demonstrated an even more pronounced reduction for transpiration (E) than for photosynthesis, indicating strong stomatal control under drought stress. The variability between genotypes is lower under stress conditions (Figure 2). The transpiration (E) parameter also showed a differential behavior in the face of drops in moisture in the soil. Under well-watered conditions, an average E value of 3.8 ± 0.6 mmol H_2_O m^−2^s^−1^ was observed. The 1451, 1433, and 1915 genotypes presented the maximum transpiration, with 4.3 ± 0.5 mmol H_2_O m^−2^s^−1^, compared to the 1424 and 1542 genotypes, with 3.0 ± 0.5 mmol H_2_O m^−2^s^−1^. In contrast, under limiting soil moisture conditions, there was a drastic decrease in the E values, with an average of 0.72 ± 0.3 mmol H_2_O m^−2^s^−1^, equivalent to a reduction of approximately 81% compared to the E values under well-watered conditions. Among the genotypes evaluated, only 1424, 1381, and 1363 had E values slightly greater than 1.0 mmol H_2_O m^−2^s^−1^, with reductions in E on the order of 57%, 71%, and 69%, respectively (Figure 2). However, the lowest transpiration values were detected for the hybrid O×G 1542 genotype (0.27 ± 0.1 mmol H_2_O m^−2^s^−1^) and the *E. oleifera* 1147 genotype (0.37 ± 0.1 mmol), which represents a reduction close to 90% in the transpiration values observed in well-watered plants.

**Figure 3:**
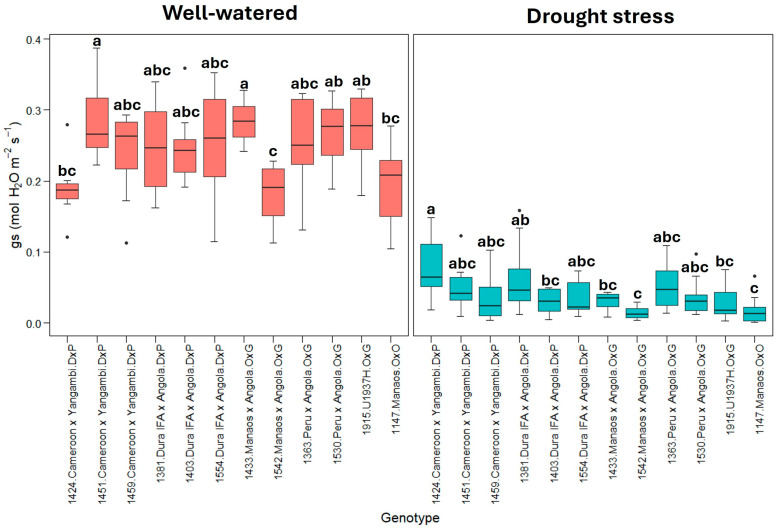
gs: stomatal conductance (mmol H_2_O m^−2^s^−1^). Treatment by genotype. Tukey’s mean comparison test shows that values with the same letter do not present statistically significant differences (*p* < 0.05).

The variable stomatal conductance (gs) (Figure 3) shows a pattern similar to transpiration, with a substantial reduction under drought stress. This finding confirms that stomatal closure is a primary response mechanism to drought stress.

**Figure 4:**
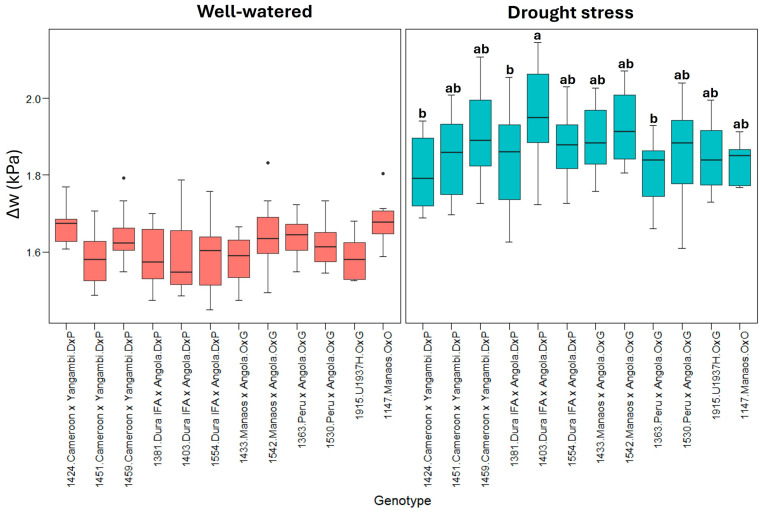
Δw: (leaf-to-air vapor pressure deficit). Treatment by genotype. Tukey’s mean comparison test shows that values with the same letter do not present statistically significant differences (*p* < 0.05). No letter for Tukey’s mean comparison test does not present statistically significant differences.

In contrast to the other variables, the difference in the leafto-air vapor pressure deficit (Δw) increased under leaf vapor pressure deficit conditions, suggesting greater evaporative demand. Variability between genotypes is more pronounced under drought stress conditions (Figure 4).

**Figure 5:**
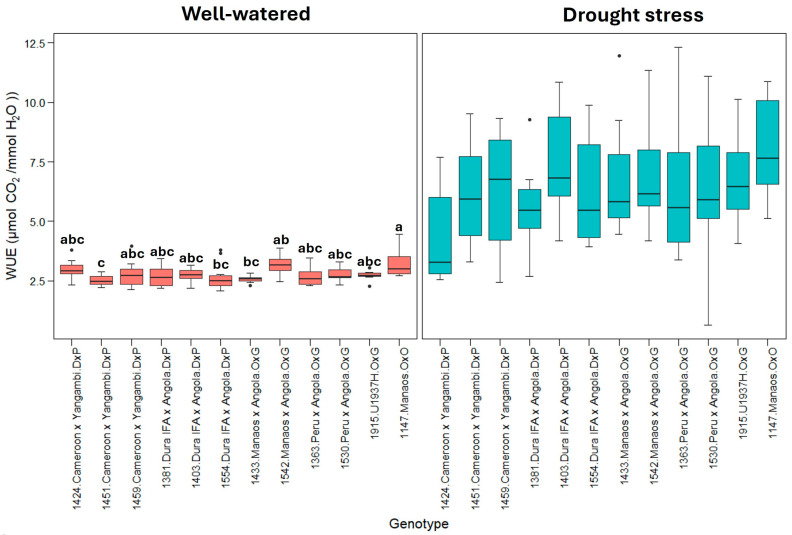
WUE: instantaneous water use efficiency (µmol CO_2_/mmol H_2_O). Treatment by genotype. Tukey’s mean comparison test shows that values with the same letter do not present statistically significant differences (*p* < 0.05). No letter for Tukey’s mean comparison test does not present statistically significant differences.

Figure 5 represents the WUE (instantaneous water use efficiency). This shows that plants under drought stress treatment use water more efficiently than well-watered plants. Similarly, the 1530_Peru × Angola genotype exhibited the highest WUE under drought stress, indicating a greater WUE under these conditions.

The instantaneous leaf-level water use efficiency (WUE) calculated during the highest gas exchange for photosynthesis and transpiration (8:30 to 11:00 h) showed that under drought stress, the stomatal opening for gas exchange was restricted in all the genotypes. The WUE variable represents the amount of µmol CO_2_ fixed by the plant during photosynthesis relative to each mmol of H_2_O lost through transpiration (A/E), and high values represent greater efficiency in the use of water for photosynthesis. Under well-watered conditions, the 12 genotypes evaluated presented an average WUE of 2.8 ± 0.3 µmol CO_2_/mmol H_2_O, especially genotypes 1424, 1542, and 1147, which showed the highest WUE, with values greater than 3.0 µmol CO_2_/mmol H_2_O (Figure 5). On the other hand, genotypes 1451 and 1433 showed the lowest efficiencies. In contrast, under drought stress conditions, the WUE values increased, reaching an average of 6.5 ± 1.6 µmol CO_2_/mmol H_2_O; however, contrary to what was observed under the optimal humidity conditions, high WUE values reflected a drastic decrease in the transpiration rate of palms subjected to stress because of stomatal closure. Therefore, genotypes with relatively high WUE values, such as 1147, 1542, and 1403, were the most sensitive to soil moisture loss regarding transpiration rate. In contrast, genotypes with relatively low WUE values, such as 1424, 1381, and 1363, exhibited better performance under stress conditions since they continue to fix CO_2_ while maintaining a relatively high transpiration rate, indicating that these genotypes retain a greater number of open stomata. 

**Figure 6:**
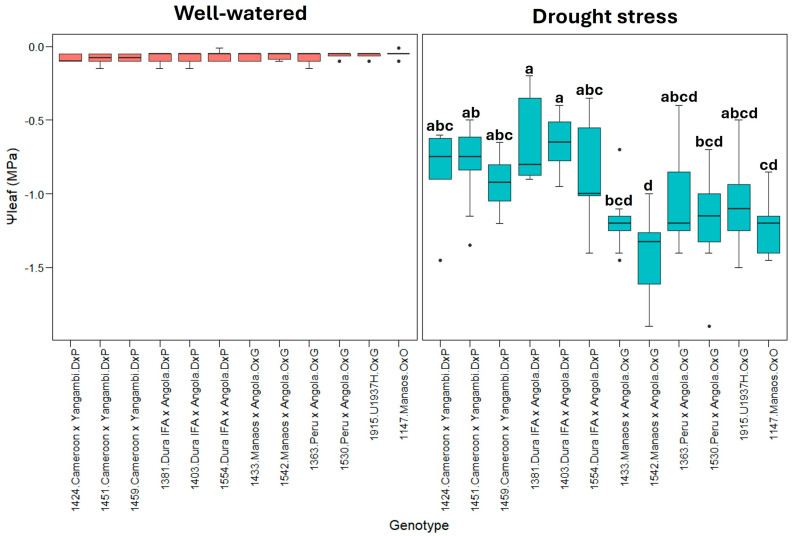
Ψleaf: Leaf water potential (bar). Tukey’s mean comparison test shows that values with the same letter do not present statistically significant differences (*p* < 0.05). No letter for Tukey’s mean comparison test does not present statistically significant differences.

Figure 6 shows the leaf water potential (Ψleaf), in which, under well-watered conditions, all the genotypes had Ψleaf values close to zero (0), indicating good water status. In contrast, a significant decrease in Ψleaf was observed under drought stress, with some genotypes reaching values as low as −1.9 MPa, indicating severe drought stress. The variability between genotypes under the two treatments suggests differences in the tolerance mechanisms to water scarcity.

For Ψleaf, the group composed of O×G hybrids and *E. oleifera* genotypes showed statistically significant differences from the *E. guineensis* genotypes in drought stress, indicating that O×G hybrids and *E. oleifera* are more sensitive to water loss (Figure 6).

Analysis of variance (ANOVA) (Table 2) revealed significant effects for each condition (well-watered and drought stress conditions) on most of the measured variables. This indicates that both conditions affected all the physiological parameters evaluated. In well-watered conditions, highly significant differences were detected between the genotypes for photosynthesis (A), transpiration (E), and stomatal conductance (gs) (*p* < 0.001). Significant differences were found for leaf–air vapor pressure deficit (Δw) (*p* < 0.05) and water use efficiency (WUE)(*p* < 0.01), showing that the genotypes evaluated respond differently to optimal irrigation conditions. In contrast, no significant differences were detected between genotypes for leaf water potential (Ψleaf), which could indicate a more uniform response of these parameters among the genotypes studied under well-watered conditions.

Under drought stress, significant differences were detected between the genotypes for photosynthesis (A) (*p* < 0.01), transpiration (E), stomatal conductance (gs), leaf water potential (Ψleaf) (*p* < 0.001), and leaf–air vapor pressure deficit (Δw) (*p* < 0.01), suggesting that the genotypes respond differently to drought stress. However, no significant differences were detected between genotypes for water use efficiency (WUE), which could indicate a more uniform response of these parameters among the genotypes studied. 

### 2.2. RT-qPCR Validation of Genes Involved in Drought Tolerance

**Figure 7:**
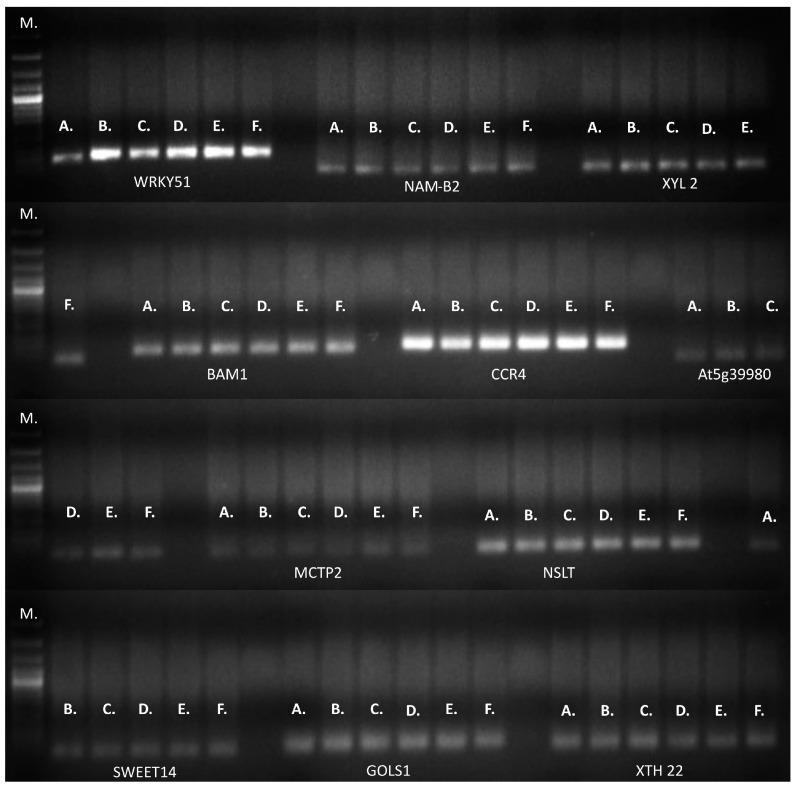
Conventional PCR of 11 genes associated with drought tolerance in a 1.5% agarose gel stained with SYBR Safe. DNA (120 ng/µL) was used as a template for each reaction. Conclusions: M: Molecular weight marker. A. 1433; B. 1530; C. 1451; D. 1147; E. 1381; F. 1915. WRKY51: WRKY transcription factor 51; NAM-B2: NAC transcription factor NAM-B2-like_NAM-B2; XYL 2: β-xylosidase α-L-arabinofuranosidase 2-like; BAM1: leucine-rich repeat receptor-like serine; CCR4: serine, threonine-protein kinase-like protein; At5g39980: pentatricopeptide repeat-containing protein; MCTP2: multiple C2 and transmembrane domain-containing protein 2-like; NSLT: nonspecific lipid-transfer protein 2-like; SWEET14: bidirectional sugar transporter SWEET14-like; GOLS1: galactinol synthase 1-like_GOLS1; XTH22: xyloglucan endotransglucosylase/hydrolase protein 22-like XTH22.

An equal amplification profile was observed for the 11 genes evaluated separately by RT-qPCR or conventional PCR (Figure 7).

## 3. Discussion

### 3.1. Physiological Parameters

The main abiotic stress factors that limit oil palm production, such as water deficit, waterlogging, and high temperatures, have been increasing due to climate change. It is also important to consider the negative impacts of controlling traditional diseases and pests and the emergence of new ones [42,43]. Genetic improvement in drought-tolerant oil palm cultivars could mitigate the effects of climate change on the crop and optimize oil palm yield [2]. These threats are also present in other crops, and each one works to improve its strategy for breeding against climate change and reaching the equilibrium between higher yields and water use efficiency [44,45].

On the other hand, a genotype’s photosynthetic capacity is directly related to its productivity, which is why the evaluation of different physiological parameters related to gas exchange proposed in the present study has become a powerful tool for identifying promising cultivars in limited water conditions.

Water deficit caused a considerable reduction in the photosynthetic capacity of the evaluated genotypes of *E. guineensis*, *E. oleifera*, and the O×G hybrids, resulting in a drastic decrease in the rates of photosynthesis and transpiration, as previously reported by different authors [9,46]. These variations are mainly due to stomatal closure, which limits the flow of CO_2_ into leaves [47].

In general, the O×G hybrids and the *E. oleifera* genotype showed greater susceptibility to changes in soil water content, particularly those that share a Manaos origin, which may be related to the environments in which it develops naturally, such as tropical rainforest margins and poorly drained areas in clay soils and savannahs [48]. Coincident with what was observed in Colombia, areas planted with O×G hybrids with better precipitation throughout the year reported the highest productivity [49]. These changes were evident in parameters such as leaf water potential and transpiration.

The genotypes Manaos × Angola 1542 and Manaos 1147 (*E. oleifera*) presented sharp decreases in stomatal conductance (gs) in drought stress. A similar result was obtained by [13], who compared the drought stress response between O×G hybrids from Manicoré (Amazon region) as a female parent and *E. guineensis* (La Mé × Deli). In our case, the comportment of O×G Manaos × Angola is different from Peru × Angola, confirming that the female origin can influence the physiological response. However, there are no similar studies that use Peru as a female parent (*E. oleifera*) origin for more comparison data.

Differences are evident in the response between the genotypes evaluated, depending on the water available in the soil. Our results show a variation in the gas exchange parameters and instantaneous water use efficiency (WUE) among the genotypes evaluated under well-watered and drought stress. Under optimal soil moisture conditions, the O×G hybrid genotypes showed a gas exchange like that registered in most *E. guineensis*. In contrast, seedlings of O×G genotypes subjected to drought stress showed a rapid drop in photosynthetic rates. Similar results were reported by [50], where drought stress for eight days generated a drastic decrease in net photosynthesis rate (A), transpiration (E), and stomatal conductance (gs) in the commercial hybrids Coari × La Mé and Amazón (Manaos × Compacta). But the most particular situation for these cultivars was that in well-watered conditions, they stood out for presenting higher values in WUE than those reported for *E. guineensis* cultivars. This situation was different from the other O×G genotypes evaluated as Taisha × AVROS and Taisha × La Mé, which did not stand out for their WUE, which confirms that each combination of the O×G hybrid deserves to be studied in a particular way when facing drought stress, thus avoiding the generalization of these results.

Under optimal conditions, the O×G 1915 (U1937H) and 1433 (Manaos × Angola) genotypes presented the highest photosynthetic rates. However, when the water availability was limited, these same genotypes showed greater sensitivity, with a more pronounced decrease in parameters related to gas exchange. In contrast, the genotypes *E. guineensis* 1424 (Cameroon × Yangambi) and 1381 (Dura IFA × Angola), which, under well-watered conditions, exhibited lower photosynthetic rates and, under drought stress, presented greater physiological plasticity by maintaining comparatively better gas exchange rates than the other genotypes.

Among the O×G hybrids, genotype 1363 (Peru × Angola) presented the lowest reduction in photosynthesis under drought stress. The O×G 1530 genotype (Peru × Angola) had one of the highest WUE values under drought stress. A high WUE physiologically indicates the trade-off between the photosynthetic carbon assimilation from carbon dioxide and the loss of water through transpiration, largely through the stomatal pores. Nevertheless, it is well known that more efficient water use does not mean greater photosynthesis, as shown in this study. The WUE at the leaf level is a complex trait dependent upon physiological variables and their responses, so more factors affect the photosynthetic process at the leaf scale [44].

According to [9], O×G U1937H reduced photosynthesis by 12.7 to 3.9 µmol CO_2_ m^−2^s^−1^ when exposed to a soil water potential of −1.0 MPa (severe water deficit) and responded successfully to drought stress (moderate and severe). In this study, our results were similar; the genotype decreased by 11.5 to 3.8, with a water potential of approximately −1.0 MPa. This genotype, U1937H, responded better to drought stress conditions than some genotypes from Manaos × Angola (Figure 1).

The results suggest that genotypes 1424 (Cameroon × Yangambi) and 1363 (Peru × Angola) can better adjust their stomatal conductance (gs) and transpiration (E), maintaining a greater CO_2_ fixation, which allows sustaining higher photosynthetic rates under drought stress (Figure 2 and Figure 3). Similar results were obtained by [47] with the *E. guineensis* cultivar IRHO7010 and by [28] with the cultivar IRHO7001, which presented a decrease in the photosynthetic rate under drought stress while maintaining a higher stomatal conductance and transpiration.

In well-watered conditions, the predawn leaf water potential (Ψleaf) values, close to zero, are related to the physiological registers of oil palm prior to drought stress conditions [7,13,28,51,52]. For this parameter (Ψleaf), both *E. guineensis* and hybrid O×G genotypes were very sensitive to water deficit, significantly reducing the values observed seven days after irrigation suspension (Figure 6). Similar results were obtained by [51], where oil palm seedlings were subjected to repetitive events of drought stress to analyze the physiological response, and where, during one week of water shortage, the Ψleaf decreased from –0.07 MPa to –1.81 MPa. In our case, the Ψleaf in drought stress has a maximum of −1.9 MPa, and the negative values are principally for hybrids O×G compared to *E. guineensis* genotypes. A similar result was reported by [13] where the values of Ψleaf decreased progressively during seven days of drought stress for *E. guineensis* and a hybrid O×G (*E. oleifera* Manicoré origin), reaching values between −1.5 MPa and −1.8 MPa, respectively. In our case, the O×G hybrids and *E. oleifera* genotypes showed statistically significant differences vs. the *E. guineensis* genotypes, which indicate that O×G hybrids are more sensitive to loss water. 

### 3.2. RT-qPCR Validation of Genes Involved in Drought Tolerance

The genes selected are closely related to protection mechanisms against drought stress and were reported in oil palm by [28]. The *SWEET* genes are involved in sugar transport from the source leaf to the sink organ in response to drought stress [30,53]. The *WRKY* transcription factor family plays essential roles in plant growth, abiotic stress responses, and defense regulation [33]. The gene expression profiles of multiple C2 domain and transmembrane region proteins increase their expression during drought stress [37], and the *GOLS1* genes, important for carbohydrate metabolism, are part of the osmoprotectant synthesis that helps maintain drought adaptation [34].

No differences were detected by RT-qPCR or conventional PCR (Figure 7) for the 11 genes validated. These results indicate that no allelic variants were represented in the RNA during sampling for validated genotypes. The above does not rule out that other variants may be found in conditions that are different from those proposed or in other genotypes. The potential development of molecular markers for M.A.S. depends on their association with yield, genetic variation, the heritability of these traits under stress, and genotype × environment interactions. The appropriate molecular marker selection is also supported by studies of the significant variation among genotypes for various morphophysiological traits over different seasons to guarantee repeatability across many crosses or genotypes.

Regarding how molecular markers are commonly used to identify associations between markers and morphophysiological traits evaluated under water stress conditions, studies on SSRs have shown their ability to separate groups by allelic polymorphisms in tolerant and moderately tolerant genotypes in wheat [54] or in maize, where the use of SSRs with polymorphic and unique alleles across genotypes was possible for discerning between tolerant and susceptible genotypes for eventual utilization in breeding programs as well as for QTL identification [55]. This method could be a good option for massive screening. Still, it is also advisable to use and know different genes that allow for the finding of different allelic variants for discrimination between populations and that could be accurate candidate genes for genetic editing.

The results showed that the *E. guineensis* genotype was the most efficient at achieving photosynthesis under drought stress conditions, followed by the hybrid and *E. oleifera* genotypes. The physiological parameters showed good prospects for vegetal breeding with different O×G hybrids, mainly because of their ability to maintain the equilibrium between CO_2_ assimilation and stomatal aperture.

In the present study, drought stress significantly affected the physiology of oil palms, as indicated by decreases in transpiration, photosynthesis, and stomatal conductance. Plants respond to water stress by closing their stomata to conserve water, limiting carbon assimilation. This response mechanism reflects a crucial adaptation of plants to survive drought conditions, although it negatively affects their growth and productivity. Furthermore, a significant increase in instantaneous water use efficiency (WUE) was observed under water deficit conditions. This indicates that plants optimize their use of resources under stress by assimilating more carbon per unit of water lost, and this increase in efficiency is an adaptive strategy that allows plants to maintain their primary metabolism and survive during periods of water scarcity.

In our case, the variability in the responses of the genotypes under water deficit, especially in WUE and leaf water potential (Ψleaf), suggests significant differences in the capacity of the genotypes to cope with water stress. Some genotypes show greater instantaneous water use efficiency or can maintain a better water status under stress conditions, which has important implications for selecting and improving oil palm genotypes with greater drought tolerance. Regarding significant differences in the response to water scarcity, variations are observed at both the species, cross, and genotype levels. 

In summary, the species *Elaeis guineensis* (D×P) and the hybrid *Elaeis oleifera* × *Elaeis guineensis* (O×G) show notable differences in their response to water deficit, with some DxP genotypes maintaining better photosynthetic rates under water stress. Similarly, the different crosses within the species show variability in their response to water stress. For example, some O×G crosses present greater water use efficiency under water deficit conditions. At the individual genotype level, there are significant differences in transpiration, photosynthesis, stomatal conductance, and leaf water potential, suggesting that specific genotypes can tolerate drought stress more than others.

These observations indicate that it is possible to select and improve specific oil palm genotypes that are more tolerant to drought. This is crucial for the sustainability of this crop under adverse climatic conditions, especially considering climate change and the increasing frequency of drought events.

## 4. Materials and Methods

### 4.1. Location 

This research was carried out at the Motilonia Research Center of AGROSAVIA located in the rural area of Agustin Codazzi, Cesar, Colombia, with the following specifications: 10°0′16.141″ N and 73°15′11.845″ W; an elevation of 100 m above sea level; an average annual rainfall of 1.585 mm; an average temperature of 28.7 °C; and an average relative humidity of 60%. The experiment was carried out in a mesh house with a 40% polyshade. Inside the mesh house, the relative humidity was lower than 50%, and the temperature reached 35 °C. To maintain the relative humidity at 50% at least and avoid limitations to photosynthesis, external humidifiers were added, which also contributed to keeping the temperature below 32 °C.

### 4.2. Plant Materials and Experimental Design

Twelve genotypes were evaluated under drought stress conditions in a mesh house. *E. guineensis* was represented by three Dura IFA × Angola genotypes and three Cameroon × Yangambi genotypes. The O×G hybrid was represented by two Manaos × Angola genotypes, two Peru × Angola genotypes, and one commercial cultivar. O×G. *E. oleifera* was represented by one genotype from Manaos (Brazil) (Table 3).

Pregerminated seeds were kept in germination soil until the plantlets presented five lanceolate leaves. Consequently, plantlets were transplanted into plastic containers with 20 kg of soil composed of 24.6% sand, 36.6% silt, and 38.7% clay, with a clay loam texture soil. The density of the soil was 1.40 g/cm^3^. Plantlets were maintained at well-watered conditions for 90 days to adapt to the new substrate. The soil water retention characteristics, including the water holding capacity and wilting point, were determined to maintain the plants under the corresponding soil water potential. This study’s water holding capacity and wilting point were approximately 27.8% and 24%, respectively.

The experimental design consisted of one randomized complete block (RCB) with two registers on the same palm. The experimental unit was composed of four plants per genotype, which were planted in four repetitions. Once the photosynthetic rate baseline was established under well-watered conditions (100% of the photosynthetic rate), the water supply was suspended for seven days, and each genotype was monitored until 50% or more of the photosynthetic rate decreased.

### 4.3. Statistical Analysis

Analysis of variance (ANOVA) was performed for each condition (well watered and drought stress). The statistical software used was R Version 4.3.0 to evaluate the physiological behavior of the 12 genotypes. Mean comparisons were made through Tukey’s Studentized Range (HSD) test. Additionally, box plots were generated to visualize the distribution of physiological parameters by genotype.

### 4.4. Physiological Parameters

To quantify gas exchange, net photosynthetic rate (A), transpiration (E), leaf–air vapor pressure deficit (Δw), and stomatal conductance (gs), the LI-6800 open-path portable photosynthesis system (LI-COR, Nebraska, EE. UU) was used. The following parameters were fixed at the following measuring points: 28 °C and 65% of the temperature and relative humidity of the chamber, an airflow rate of 300 μmol s^−1^, a CO_2_ concentration in the chamber of 400 ppm, and a PAR of 1000 μmol photons m^−2^s^−1^. The measurements were taken on the third leaf of the palms in the morning between 9:00 and 11:00. To verify whether the plants were stressed, the instantaneous leaf water use efficiency (WUE) was determined by the ratio of A to E (A/E). The leaf water potential (ΨLeaf) was determined under drought stress and well-watered conditions using a Plant Water Status Console device, Model 3005 (Soilmoisture Equipment, Santa Barbara, CA, USA), between 4:00 and 6:00 h (predawn). The gas exchange measurements were made in four plants by code by repetition.

### 4.5. RT-qPCR Validation of Genes Involved in Drought Tolerance

To perform gene expression validation by RT-qPCR and conventional PCR, leaf tissue samples under drought stress conditions were stored in NucleoProtect RNA (Macherey-Nagel. Ref 740400.250) to preserve RNA integrity before storage at −80 °C. To verify whether there were differentially expressed genes among the genotypes of *E. guineensis*, the O×G hybrid, and *E. oleifera*, 11 genes found via the transcriptome analysis of oil palm plants under drought stress [28] or in response to abiotic stress in different crops were selected (Table 4). qPCR was performed in a 10 µL reaction mixture using Fast Evagreen^®^ qPCR Master Mix (Biotium, Inc., Fremont, CA, USA) in a real-time QIAquant 96 5plex (Qiagen, Germantown, MD, USA) following the manufacturer’s instructions. The relative expression of each gene was calculated using the delta-delta Ct method (2^^∆∆Ct^), and the GAPDH and EF1 genes were utilized as normalizers. All PCRs started with 120 ng of DNA.

## Figures and Tables

**Table 1:** Descriptive statistics by treatment. A: net photosynthetic rate (µ mol CO_2_ m^−2^s^−1^), E: transpiration rate (mmol H_2_O m^−2^s^−1^), gs: stomatal conductance (mmol H_2_O m^−2^s^−1^), Δw: (leaf-to-air vapor pressure deficit), WUE: instantaneous water use efficiency (µmol CO_2_/mmol H_2_O), Ψleaf: leaf water potential (MPa). Each number corresponds to the mean ± SD.

Treatment	A	E	gs	Δw	WUE	Ψleaf
Well-watered treatment	10.4 ± 1.37 *	3.84 ± 0.84	0.24 ± 0.06	1.61 ± 0.08	2.79 ± 0.45	−0.07 ± 0.03
Drought stress	3.80 ± 2.03	0.71 ± 0.56	0.03 ± 0.03	1.87 ± 0.11	6.49 ± 2.41	−1.0 ± 0.36

* mean ± sd.

**Table 2:** ANOVA. A: net photosynthetic rate (µ mol CO_2_ m^−2^s^−1^), E: transpiration rate (mmol H_2_O m^−2^s^−1^), gs: stomatal conductance (mmol H_2_O m^−2^s^−1^), Δw: (leaf-to-air vapor pressure deficit), WUE: leaf water use efficiency to photosynthesis (µ mol CO_2_/mmol H_2_O), Ψleaf: leaf water potential (MPa).

Condition	Parameter	Sum Sq	Mean Sq	F Value	Pr (>F) *
Well-watered	A	66.42	6038	4.251	3.63 × 10^−5^ ***
	E	22.69	20631	3.805	0.000143 ***
	gs	0.12271	0.011155	3.657	0.000227 ***
	Δw	0.1289	0.011719	2.229	0.01843 *
	WUE	5.630	0.5118	2.940	0.0021 **
	Ψleaf	0.00692	0.0006291	0.755	0.6838
Drought stress	A	109.3	9.932	3.009	0.00172 **
	E	9.885	0.8986	3.652	0.000234 ***
	gs	0.03251	0.0029550	3.661	0.000228 ***
	Δw	0.1998	0.01816	2.931	0.00218 **
	WUE	80.3	7.301	1.255	0.262
	Ψleaf	6.139	0.5581	7.139	8.63 × 10^−9^ ***

Statistical significance levels: * *p* ≤ 0.05. ** *p* ≤ 0.01. *** *p* ≤ 0.001.

**Table 3:** List of the species, genetic origin of the parental line, and genotypes subjected to drought stress.

Species/Genotype	Female Parent	Male Parent	Code Genotype
*Elaeis oleifera (O×O)*	Manaos	Manaos	1147
*Elaeis guineensis (D×P)*	Dura IFA	Angola	1381
			1403
			1554
	Cameroon	Yangambi	1424
			1459
			1451
*E. oleifera × E. guineensis* (O×G)	Peru	Angola	1530
			1363
	Manaos	Angola	1433
			1542
	*E. oleifera*	Congo Mixto	1915(U1937H)

**Table 4:** Selection of genes for validation by RT-qPCR and conventional PCR.

Anotation	5’→3’	Sequence	Tm	Expected Size (pb)	References
WRKY transcription factor 51	F	TTATGTGAAGACCGACCCATC	59.4	140	[32]
	R	CAGGACAGGAAGGAGCAAAG	60.5		
NAC transcription factor NAM-B2-like_ NAM-B2	F	CGTCCTCTCACTTCCCCATAC	63.3	127	[35]
	R	CGCTGCTGGTGTTGTTGTT	57.3		
beta-xylosidase alpha-L-arabinofuranosidase 2-like OsI_08964_ XYL2	F	CTTCAAACCCATCCACCAAG	58.4	128	[41]
	R	AGGGGCACTATCACCCAGTT	60.5		
Leucine-rich repeat receptor-like serine_ At1g17230	F	CATCATCACAAGCACAAGCA	56.4	121	[22]
	R	TCTCCAAGAATGCGAGGTGT	58.4		
Multiple C2 and transmembrane domain-containing protein 2-like	F	GGATACAGACGGTGGTAGGG	62.5	105	[36]
	R	GGCAGAACATCACAAACAGG	58.4		
Nonspecific lipid-transfer protein 2-like	F	GTCCTCTGCTTGCTGTGCTA	60.5	112	[23,40]
	R	AACCACTTTCTGGCTGTTGG	58.4		
Pentatricopeptide repeat-containing protein_At5g39980	F	AGCGATGTCTCAGTGCTTCTC	61.3	94	[39]
	R	CTTCCTCCTGTTCCTCTCCA	60.5		
Serine, threonine-protein kinase-like protein_CCR4	F	ACAGCACCAGCAAGGAGAGA	60.5	91	[38]
	R	GCTTTGAGGAGGGTTTCCA	57.3		
Bidirectional sugar transporter SWEET14-like	F	AACGTGGTGCTATTCGGGTT	61.1	93	[29]
	R	ACTCACACAGACCCATCCAAG	62.4		
Galactinol synthase 1-like_ GOLS1	F	TGCGGTGATGGATTGCTTCT	61.2	142	[35]
	R	ACCAGCGTTGAAGTACAGGG	62.1		
Xyloglucan endotransglucosylase/hydrolase protein 22-like XTH22	F	TCCTCTGCTTCCCCTCTACC	65.2	120	[31]
	R	CGCCCCAAGTGATCTGGAAA	63.6		
Glyceraldehyde-3-phosphate dehydrogenase GAPDH	F	CAACCAACTGTCTTGCTCCTT	59.4	138	[25]
	R	CTCCTCGCCAATCTTTCATT	56.4		
Elongation factor 1-alpha_EF1-a1	F	CCTTCTTGCTTTCACCCTTG	58.4	143	[31]
	R	GGTTGTAGCCGACCTTCTTG	60.5		

## Data Availability

The data presented in this study are available upon request from the corresponding author. Due to privacy restrictions, they are not publicly available.
